# Evaluation of a Meds-to-Beds Program to Reduce Readmissions After Percutaneous Coronary Intervention

**DOI:** 10.1016/j.jacadv.2026.102611

**Published:** 2026-03-25

**Authors:** Katherine E. Di Palo, Pavel Goriacko, Mark Menegus, E Scott Monrad, Nicholas J. Barone, Mark Sinnett, Angela Cheng-Lai

**Affiliations:** a Montefiore Health System, Center of Pharmacotherapy Research & Quality, Bronx, New York, USA; b Albert Einstein College of Medicine, Department of Medicine, Bronx, New York, USA; c Albert Einstein College of Medicine, Department of Epidemiology & Population Health, Bronx, New York, USA

**Keywords:** hospital readmissions, meds-to-beds, percutaneous coronary intervention, pharmacist, transitions of care

## Abstract

**Background:**

Unplanned hospitalizations following percutaneous coronary intervention (PCI) are common and represent an opportunity to improve outcomes and reduce costs. However, health system interventions to reduce readmissions in this population are not well described.

**Objectives:**

We aimed to implement a meds-to-beds program and evaluate its impact on 30-day readmissions.

**Methods:**

We performed an observational study among a medically underserved diverse cohort of adults who received emergent or elective PCI at an academic medical center in Bronx County, NY. The meds-to-beds program consisted of a pharmacist-directed counseling and a free, 1-month supply of dual antiplatelet therapy before discharge. The intervention was compared to the standard of care control group that received instructions and dual antiplatelet therapy prescriptions at discharge. The primary outcome was 30-day all-cause hospital readmissions. Secondary outcomes included 30-day cardiac-related readmissions and provider satisfaction.

**Results:**

A total of 662 patients were included. In a multivariable analysis, 30-day all-cause readmissions did not differ between the intervention and control groups with respective incidences of 9.0% and 9.7% (OR: 1.07; 95% CI: 0.58-1.97). Although the 30-day cardiac-related readmission rate was significantly lower in the intervention group (2.9%) compared to the control group (6.3%), this association did not remain significant after confounder adjustment (OR: 0.50; 95% CI: 0.21-1.18). Providers perceived that the intervention improved safety and quality during transitions of care.

**Conclusions:**

A meds-to-beds program was not associated with readmission reduction among adults who received PCI. Other measures, such as medication adherence and patient satisfaction, could be employed to assess the effectiveness of future medication delivery programs.

Within the current value-based care landscape, 30-day all-cause hospital readmissions are a proxy metric for transitional care quality and represent an opportunity to curtail health care costs and improve patient outcomes. Unplanned readmissions following percutaneous coronary intervention (PCI) are common with rates ranging from 6% to 17%.^1^ Risk factors for rehospitalization are well described and include female sex, Medicare insurance, advanced age, acute coronary syndrome presentation, baseline comorbidities, and receiving an oral anticoagulant at discharge.^2^, ^3^, ^4^, ^5^, ^6^ Despite data suggesting that nearly half of PCI readmissions may be preventable,^7^ there is a paucity of evidence-based health system interventions to reduce 30-day readmission rates. Data-driven strategies specifically designed for socially vulnerable and diverse populations are even more scarce.

Although medication-related issues are a common driver of rehospitalization, barriers specifically to dual antiplatelet therapy (DAPT) are a safety concern among patients who receive PCI with stent-revascularization, given the risk for stent thrombosis. However, up to one-third of patients may not comprehend the need or have the means to acquire all of their new medications within 30 days after discharge following cardiac catheterization^8^ Furthermore, patients who have delays in filling their medications were found to have higher 30-day and 1-year mortality rates when compared to patients who obtained all of their medications in a timely manner.^9^^,^^10^ Health system and clinician-related drivers of nonadherence, including prior authorization, adequate time for education and counseling, and accurate discharge summaries, are modifiable especially through team-based care.^11^ Meds-to-beds, which includes the delivery of postdischarge medications to patients before they leave the hospital, may alleviate these causes of nonadherence and ensure access in high-risk patients with social needs. In a modified stepped wedge trial across 15 hospitals, a multifaceted intervention, including bedside medication delivery, education, refill reminders, and outreach, significantly reduced delays in filling prescriptions and improved 1-year adherence after PCI.^12^ To date, there have been mixed results of these specific transitions of care interventions on readmission reduction.^13^, ^14^, ^15^, ^16^ The purpose of this study is to assess the impact of a comprehensive meds-to-beds program on 30-day readmissions and to evaluate provider perception and satisfaction.

## Methods

### Study design

This was a retrospective, observational study of adults aged 18 years of age or older who received PCI at an urban, multicampus academic medical center in Bronx County, New York City between 2016 and 2017 with follow-up for at least 1 year after discharge. Patients were included if they underwent PCI during hospitalization, regardless of procedural complexity or completeness of revascularization, and were prescribed specific DAPT regimens (aspirin and clopidogrel or ticagrelor) at discharge. Patients who were prescribed aspirin and prasugrel or expired during index admission were excluded. The meds-to-beds intervention was triggered by referral from an interventional cardiologist or physician assistant and encompassed a free 30-day supply of DAPT delivered to the bedside coupled with standardized pharmacist-directed education on medication indication, administration, duration, side effects, and adherence. The health system assumed the cost of the meds-to-beds program, including the 30-day supply of DAPT and pharmacist counseling. Patients incurred no out-of-pocket costs, and no professional or facility fees were billed for participation in the program. The meds-to-beds program was compared to the standard of care control group which consisted of DAPT prescriptions sent electronically to the patient’s preferred community pharmacy for pick-up after discharge and nurse-led discharge instructions. Comorbidity and demographic data, including self-reported race and ethnicity, was extracted from the electronic health record. Analyses were restricted to patients with complete data. The study was approved by the Institutional Review Board as a minimum risk study with a waiver of informed consent and followed the Reporting of Studies Conducted Using Observational Routinely Collected Health Data (RECORD) reporting guideline,^17^ an extension of the Strengthening the Reporting of Observational Studies in Epidemiology (STROBE) reporting guideline.

### Outcome measures

The primary aim of this study was to evaluate the impact of the meds-to-beds program on the 30-day all-cause readmission rate following PCI. Unplanned, emergent readmissions and deaths were identified within the electronic health record, categorized by International Classification of Diseases-10 codes, and limited to hospitals within the health system. Readmissions for planned procedures, including staged PCI, were excluded from analysis. Secondary aims included 30-day cardiac-related readmissions including clinical events related to PCI (eg, in-stent thrombosis, hematoma), provider satisfaction with meds-to-beds implementation, and provider perception of the program in terms of quality and safety. Mortality at 1-year post-PCI was also examined as an exploratory endpoint. To evaluate provider perceptions of feasibility and perceived value of the program, a 6-question survey was constructed using a 5-point Likert scale (“strongly agree” to “strongly disagree”) along with an optional free-text field for qualitative feedback regarding experience. The survey was distributed electronically via email to all interventionalists, cardiologists, physician assistants, and cardiology fellows involved in PCI care during the study period and included a screener question to determine if the provider had referred patients to the program.

### Statistical analysis

Demographic characteristics between groups were analyzed using a chi-square test or Fisher exact test for categorical data and t-test or Mann-Whitney U test for continuous data. The primary endpoint, defined as the odds of 30-day readmission between intervention and control groups, was analyzed using bivariable logistic regression. Confounder adjustment was performed by specifying a multivariable logistic regression model which included intervention vs control group as all potential confounding variables as predictors. The secondary endpoint of cardiac-related readmissions was analyzed using the same methodology. Survey responses were summarized into 3 categories (“strongly agree/agree”, “neutral”, and “strongly disagree/disagree”).

### Confounder selection

Due to the nonrandomized design of the study, the investigators conducted a priori assessment of the most likely confounders that could bias the primary endpoint estimates using subject matter expertise and standard differences at baseline between groups. Potential confounders were defined as variables that could plausibly affect the provider's decision to refer a patient to pharmacist counseling and simultaneously could influence the readmission rate. The investigators selected the likely confounders to be acute coronary syndrome presentation, hospital campus, discharge antiplatelet therapy, current smoking status, diabetes diagnosis, admission type, prior cardiac history (PCI or myocardial infarction [MI]), prior DAPT therapy, and LACE index. The length of stay (“L”); acuity of the admission (“A”); comorbidity of the patient (measured with the Charlson comorbidity index score) (“C”); and emergency department use (measured as the number of visits in the six months before admission) (“E”) to estimate the risk of 30-day readmission or death.^18^

### Sensitivity analysis

As a sensitivity analysis, we performed propensity score matching to estimate the association between cohort and 30-day readmission rates (all-cause and cardiac). Propensity scores were calculated using logistic regression with the same covariables included in the primary multivariable model. We used 1:1 optimal matching without replacement, applying a caliper of 0.1 times the SD of the logit of the propensity score (Supplemental Table 1). Adequate covariable balance was defined a priori as standardized mean differences < 0.2 for all covariables. The OR for cohort was estimated using logistic regression in the matched sample with robust SEs clustered by matched pair.

## Results

### Baseline characteristics

A total of 662 patients were included; 343 were referred to the meds-to-beds intervention and 319 received the standard of care (Supplemental Figure 1). Over 90% of PCIs in both groups were emergent. There were no significant differences in stent type, current smoking status, body mass index or length of stay (Table 1). Compared to the intervention group, patients in the control group were older (66.1 years vs 63.6 years) and more frequently self-identified as Hispanic (31.4% vs 20.4%). They also had a higher prevalence of diabetes, history of MI or PCI, and correspondingly higher rate of prior DAPT therapy. Ticagrelor was prescribed more frequently to patients in the intervention group (25.4% vs 11.0%) who were more frequently admitted for a STEMI and discharged from Campus 1.

**Table 1:** Baseline Characteristics

	Intervention (n = 343)	Control (n = 319)	*P* Value
Age, y, mean (SD)	63.6 (12.0)	66.1 (12.3)	0.005
Sex, n (%)			0.842
Female	119 (34.7)	114 (35.7)	
Male	224 (65.3)	204 (64.3)	
Race, n (%)			0.138
Black	53 (15.5)	51 (16.0)	
White	71 (20.7)	90 (28.2)	
Asian	10 (2.9)	7 (2.2)	
Native American	1 (0.3)	0 (0)	
Race not identified	208 (60.6)	171 (53.6)	
Ethnicity, n (%)			<0.001
Hispanic	70 (20.4)	100 (31.4)	
Non-Hispanic	147 (42.9)	159 (49.8)	
Ethnicity not identified	126 (36.7)	60 (18.8)	
Hospital campus, n (%)			<0.001
Campus 1	298 (86.9)	194 (60.8)	
Campus 2	45 (13.1)	125 (39.2)	
Prior antiplatelet therapy, n (%)	68 (19.8)	127 (39.8)	<0.001
Discharge antiplatelet regimen, n (%)			<0.001
Clopidogrel + aspirin	256 (74.6)	284 (89.0)	
Ticagrelor + aspirin	87 (25.4)	35 (11.0)	
Clinical indication for PCI, n (%)			<0.001
NSTEMI	77 (22.5)	78 (24.4)	
STEMI	122 (35.6)	42 (13.2)	
UA	144 (41.9)	199 (62.4)	
Previous PCI, n (%)	100 (29.2)	150 (47.0)	<0.001
Previous MI, n (%)	85 (24.8)	118 (37.0)	0.001
Stent type			0.685
DES	312 (90.9)	296 (92.8)	
BMS	14 (4.1)	10 (3.1)	
None	17 (5.0)	13 (4.1)	
Current smoker, n (%)	69 (20.1)	54 (16.9)	0.340
Diabetes, n (%)	155 (45.2)	183 (57.4)	0.002
Admission type, n (%)			0.064
Emergent	322 (93.9)	310 (97.2)	
Elective	21 (6.1)	9 (2.8)	
Charleson comorbidity index, mean (SD)	2.6 (1.9)	3.0 (1.9)	0.004
BMI (kg/m^2^), mean (SD)	28.9 (5.8)	28.8 (6.0)	0.769
LACE index, mean (SD)	9.1 (2.9)	9.9 (3.3)	0.002
Length of stay, days, mean (SD)	3.4 (1.4)	3.4 (1.6)	0.762

BMI = body mass index; BMS = bare-metal stent; DES = drug-eluting stent; LACE = length of stay (“L”); acuity of the admission (“A”); comorbidity of the patient (measured with the Charlson comorbidity index score) (“C”); and emergency department use (measured as the number of visits in the six months before admission) (“E”); MI = myocardial infarction; NSTEMI = non–ST-elevated myocardial infarction; PCI = percutaneous coronary intervention; STEMI = ST-elevated myocardial infarction; UA = unstable angina.

### 30-day readmission rate and 1-year mortality

There was no significant difference in the 30-day unadjusted all-cause hospital readmission rate between patients who received the meds-to-beds intervention and those who received the standard care, with rates of 9.0% and 9.7%, respectively (OR: 0.92; 95% CI: 0.55-1.56). In the adjusted analysis, the intervention remained nonsignificant for all-cause readmissions (OR: 1.07; 95% CI: 0.58-1.97). The 30-day cardiac-related readmission rate was significantly lower in the intervention group (2.9%) compared to the control group (6.3%) in the unadjusted analysis (OR: 0.45; 95% CI: 0.20-0.95). In the adjusted analysis, the effect estimate was similar but no longer statistically significant (OR: 0.50; 95% CI: 0.21-1.18).

In the propensity score-matched sensitivity analysis, the findings were consistent with the primary adjusted analysis. For all-cause 30-day readmission, the OR was 1.00 (95% CI: 0.49-2.04; *P* = 0.99), and for cardiac-related 30-day readmission, the OR was 0.49 (95% CI: 0.15-1.40; *P* = 0.18). Adequate covariable balance was achieved across all matched variables (all standardized mean differences <0.2) (Supplemental Table 1).

Within 1 year of discharge, there were 9 deaths in the control group (2.8%) and 1 death in the intervention group (0.3%).

### Provider satisfaction and perception

The survey was distributed to 24 providers and 19 responses were received which included 7 cardiologists, 6 interventionalists, 5 physician assistants, and 1 cardiology fellow (Table 2). The majority of providers “strongly agreed” or “agreed” that the referral process was easy to follow (84%), well-integrated into discharge planning (78%), and improved quality during transitions of care (84%). Most providers “strongly agreed” or “agreed” that their patients benefited from medication delivery (89%) and pharmacist counseling (84%) before discharge. All providers “strongly agreed” or “agreed” that the PCI meds-to-beds program improved patient safety.

**Table 2:** Provider Satisfaction and Perception Survey Responses

Survey Response (n = 19)	Satisfaction Rating, n (%)		
	Strongly Agree/Agree	Neutral	Strongly Disagree/Disagree
The referral process for the meds-to-beds program was easy to follow	16 (84)	0	3 (16)
The meds-to-beds program was well-integrated into discharge planning	15 (78)	2 (11)	2 (11)
The meds-to-beds program improved quality during transitions of care	16 (84)	3 (16)	0
The meds-to-beds program improved patient safety	19 (100)	0	0
My patients benefited from medication delivery prior to discharge	17 (89)	0	2 (11)
My patients benefited from pharmacist counseling prior to discharge	16 (84)	1 (5)	2 (11)

## Discussion

In this observational study, a meds-to-beds program complemented by pharmacist-directed medication education was not associated with a reduction in 30-day all-cause or cardiac-related readmissions among patients who received PCI (Central Illustration). Our findings contrast with a similar intervention which noted a reduction in 30-day readmissions among a small cohort of patients who received medication counseling by a pharmacist during hospitalization for an acute MI.^19^ However, this intervention included a postdischarge phone call which may have further reinforced medication adherence or identified nonmedication-related postdischarge issues resulting in readmission avoidance.

**Central Illustration:**
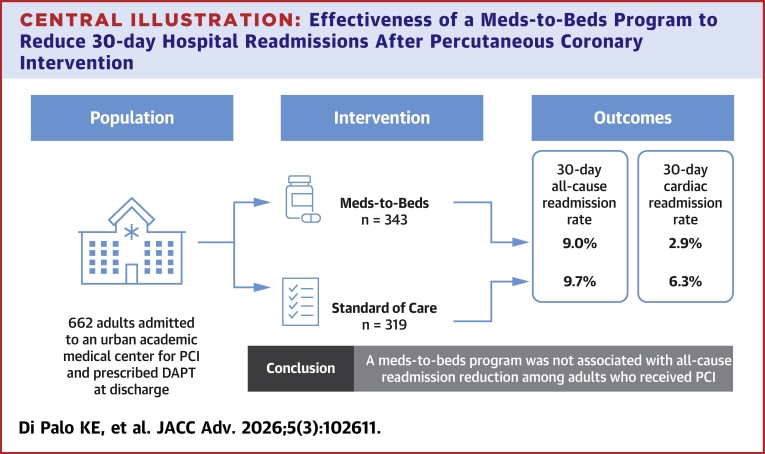
**Effectiveness of a Meds-to-Beds Program to Reduce 30-day Hospital Readmissions After Percutaneous Coronary Intervention** Overview of a meds-to-beds intervention following percutaneous coronary intervention. The program includes bedside delivery of a 30-day supply of dual antiplatelet therapy and standardized pharmacist-directed education addressing medication indication, administration, duration, side effects, and adherence. The intervention is compared with standard discharge care in which prescriptions are sent electronically to a community pharmacy for pickup after discharge.

Our study highlights the difficulties in evaluating and interpreting the effectiveness and generalizability of hospital-based readmission reduction efforts on a local scale. Although 30-day all-cause readmissions are an important quality measure, it is unlikely that a single intervention can drive the readmission rate endpoint by more than a few percent points. In addition, it may not be feasible or practical to isolate a test of change in a real-world setting. Although our meds-to-beds program may have improved other clinically relevant measures, such as stent thrombosis rates, detecting a reduction would require a substantially larger sample size due to low event rates. This makes it challenging to differentiate a truly ineffective program from a moderately effective program that is evaluated in an underpowered sample. Therefore, interventions like meds-to-beds should not be isolated and instead be incorporated into more holistic, comprehensive approaches to transitional care.

Future meds-to-beds evaluations should therefore focus on more proximal measures of implementation fidelity and effectiveness including longitudinal medication adherence, patient-reported outcomes regarding discharge experience and medication comprehension, and safety. This is especially relevant in the context of our provider survey findings. All respondents perceived that the meds-to-beds program improved safety and the majority perceived that it improved care transitions and benefited their patients. Although exploratory, 1-year mortality among persons who received the intervention was 0.3% compared to 2.8% in the control group. These findings are consistent with a post hoc analysis from a multicenter trial, which showed lower rates of MI, stroke, and death among intervention patients who received education and provision of a P2Y_12_ inhibitor at the bedside before discharge.^12^ Furthermore, more specific readmission endpoints, such as cardiac-related instead of all-cause readmissions, should be used in future evaluations. Our study provides important baseline cardiac-related readmission rates (6.3% in the control group vs 2.9% in the intervention group) that can be used to estimate sample sizes required for a thorough evaluation of this clinical effectiveness endpoint.

### Study Limitations

Our study is subject to several limitations. The meds-to-beds program was implemented within routine discharge workflows to reduce readmissions. As such, outcomes reflect real-world practice rather than a controlled experimental setting. Although multiple discharge processes were in place during the study period, including nurse-led telephonic assessment and expedited cardiology follow-up, the objective was to evaluate the incremental impact of the intervention embedded in usual care. The intervention was also triggered by provider referral which may represent selection bias toward patients who have a history of nonadherence or other social vulnerabilities that can lead to rehospitalization. However, the meds-to-beds service was contingent on pharmacy operational capacity at the time of discharge rather than purposeful provider selection. The program was initially launched at 1 campus and later expanded to a second, larger campus with higher patient volume and competing pharmacy responsibilities. As a result, a substantial proportion of eligible patients were not referred due to practical limitations in medication delivery and bedside education. This real-world constraint likely contributed to the observed imbalance between groups. Our survey results revealed that a minority of providers did not perceive benefit from pharmacist-driven counseling or medication delivery before discharge and strongly disagreed that meds-to-beds process was easy to follow and well integrated into discharge planning. These findings also indicate possible disparities in multisite program implementation which should be tracked in future real-world interventions.

Finally, we did not assess primary medication nonadherence in the control group (ie, the number of patients who did not obtain DAPT after discharge) or subsequent secondary nonadherence among patients who obtained their medication in either group. To this end, targeted provision of counseling and medication coordination toward patients with documented nonadherence or significant barriers to obtaining medications may maximize the effectiveness of a meds-to-beds program. Previous research in our institution has demonstrated that unmet social needs, particularly lack of access to health care transportation, was strongly associated with appointment no-show rates.^20^ Similar challenges are likely to drive medication nonadherence, and therefore targeted provision of medications toward patients who screen positive for social determinants of health can likely yield high clinical impact.

## Conclusions

Implementation of a meds-to-beds pharmacist-directed education program was not associated with a reduction in 30-day all-cause or cardiac-related readmissions among a diverse cohort of patients who received PCI. Tailoring the intervention toward patients with a history of nonadherence or barriers to medication access may enhance effectiveness. Other outcome measures, including patient satisfaction and experience, should also be employed to assess the utility of future medication delivery programs.

Perspectives **COMPETENCY IN SYSTEMS-BASED PRACTICE:** Unplanned readmissions after PCI underscore the need for coordinated transitional care models that integrate medication management, patient education, and structured follow up to support safe recovery after discharge.

**TRANSLATIONAL OUTLOOK:** Future efforts should focus on targeting meds-to-beds interventions to patients at highest risk for nonadherence and embedding pharmacy-led discharge strategies within scalable multidisciplinary care pathways to improve post PCI outcomes.

## Funding support and author disclosures

The project described was supported by the National Center for Advancing Translational Sciences (NCATS), National Institutes of Health, through CTSA award number UM1TR004400. The authors have reported that they have no relationships relevant to the contents of this paper to disclose.
