# Supplementary material for: Evaluation of a Meds-to-Beds Program to Reduce Readmissions After Percutaneous Coronary Intervention
Source: JACC Adv. 2026 Mar 25;5(3):102611. doi: 10.1016/j.jacadv.2026.102611 (PMC13352033; doi:10.1016/j.jacadv.2026.102611)

Supplemental Table 1. Baseline Characteristics After Propensity Score Matching

|  | Control (n=180) | Intervention (n=180) | Standardized mean difference |
| --- | --- | --- | --- |
| Age, years, mean (SD) | 66.7 (12.7) | 66.5 (12.6) | 0.03 |
| Sex, Female, n (%) | 67 (37.2) | 65 (36.1) | 0.02 |
| Race, n (%)   - Black - White - Asian - Race not identified | \| 57 (31.7) \| \| --- \| \| 27 (15.0) \| \| 5 ( 2.8) \| \| 91 (50.6) \| | \| 45 (25.0) \| \| --- \| \| 28 (15.6) \| \| 7 ( 3.9) \| \| 100 (55.6) \| | 0.156 |
| Ethnicity, n (%)   - Hispanic - Non-Hispanic - Ethnicity not identified | \| 39 (21.7) \| \| --- \| \| 97 (53.9) \| \| 44 (24.4) \| | \| 41 (22.8) \| \| --- \| \| 87 (48.3) \| \| 52 (28.9) \| | 0.119 |
| Hospital campus, Campus 1, n (%) | 159 (88.3) | 158 (87.8) | 0.01 |
| Prior antiplatelet therapy, n (%) | 57 (31.7) | 56 (31.1) | 0.01 |
| Discharge antiplatelet regimen, n (%)   - Clopidogrel + aspirin - Ticagrelor + aspirin | 134 (74.4)  46 (25.6) | 135 (75.0)  45 (25.0) | 0.01  0.01 |
| Clinical indication for PCI, n (%)   - NSTEMI - STEMI - UA | 46 (25.6)  37 (20.6)  97 (53.9) | 45 (25.0)  37 (20.6)  98 (54.4) | 0.01  0.01  <0.01 |
| Previous PCI, n (%) | 71 (39.4) | 70 (38.9) | 0.01 |
| Previous MI, n (%) | 63 (35.0) | 64 (35.6) | 0.01 |
| Stent type   - DES - BMS - None | 169 (93.9)  5 (2.8)  6 (3.3) | 170 (94.4)  4 (2.2)  6 (3.3) | 0.02  0.04  <0.01 |
| Current smoker, n (%) | 41 (22.8) | 42 (23.3) | 0.01 |
| Diabetes, n (%) | 100 (55.6) | 98 (54.4) | 0.02 |
| Admission type, Emergent, n (%) | 175 (97.2) | 176 (97.8) | 0.04 |
| Charleson comorbidity index, mean (SD) | 9.53 (3.09) | 9.53 (3.11) | <0.001 |
| BMI (kg/m^2^), mean (SD) | 28.3 (4.9) | 28.5 (5.0) | 0.04 |
| LACE index, mean (SD) | 9.5 (3.1) | 9.4 (3.0) | 0.03 |
| Length of stay, days, mean (SD) | 5.11 (5.02) | 4.63 (3.79) | 0.107 |

ACS, acute coronary syndrome; BMI, body mass index; BMS; bare-metal stent; DES, drug-eluting stent, ED, emergency department; NSTEMI, non ST-elevated myocardial infarction; PCI, percutaneous coronary intervention; SD, standard deviation; STEMI, ST-elevated myocardial infarction; UA, unstable angina

Supplemental Figure 1.
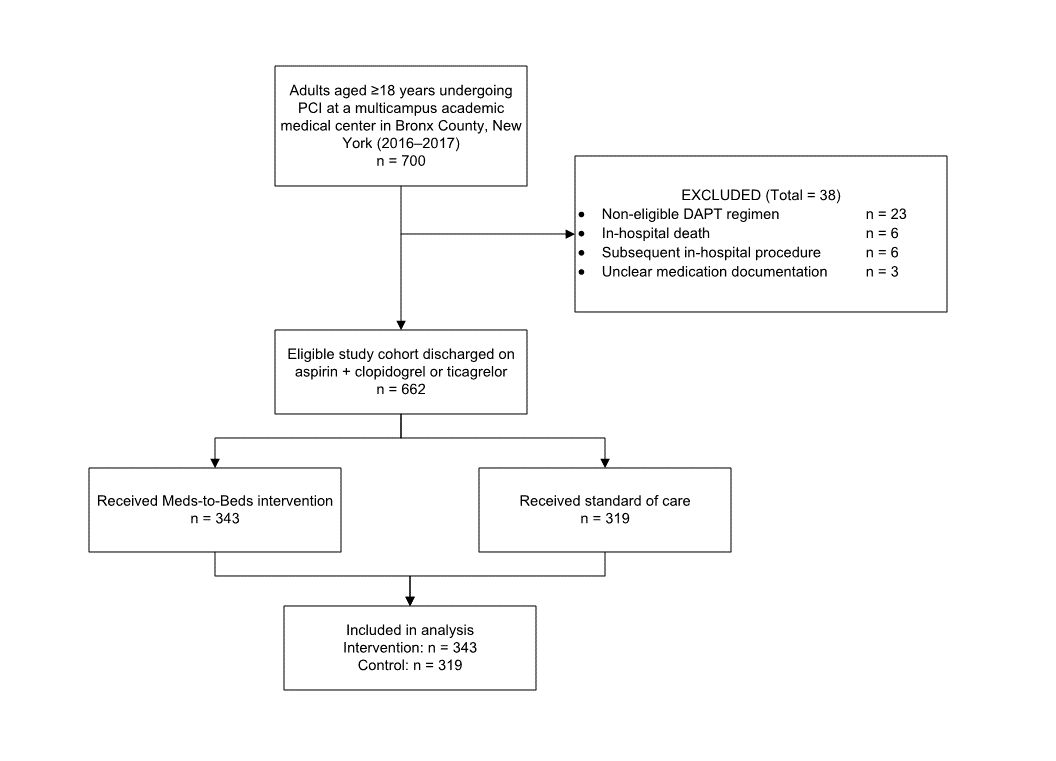

Supplement: Supplemental Material [file mmc1.docx]
